# Genome Characterization of a Novel Hepe-like Virus and a Rhabdovirus Identified in *Macrosteles fascifrons*

**DOI:** 10.3390/insects17050479

**Published:** 2026-05-08

**Authors:** Danfeng Ge, Zhi Ni, Jingya Wang, Qianqian Li, Yuting Jia, Xinyu Wei, Chuanhao Hu, Ruijun Fan, Wangxing Yang, Shishuai Lin, Zhiyuan Wu, Renyi Liu, Jiajing Xiao

**Affiliations:** 1 College of Life Sciences, Haixia Institute of Science and Technology, Fujian Agriculture and Forestry University, Fuzhou 350002, China; dfge@fafu.edu.cn (D.G.); nz1584378052@163.com (Z.N.); wangjya591@163.com (J.W.); averyever@163.com (Q.L.); 15813967726@163.com (Y.J.); hu212645935@163.com (C.H.); fanruijun20@163.com (R.F.); 13229789665@163.com (S.L.); 2 Fujian Key Laboratory of Crop Genetic Improvement and Innovative Utilization for Mountain Area, Sanming Academy of Agricultural Sciences, Sanming 365509, China; wxy1209@163.com (X.W.); yyy99425@163.com (W.Y.); 3 Rice Research Institute, Fujian Academy of Agricultural Sciences, Fuzhou 350019, China

**Keywords:** *Macrosteles fascifrons*, *Rhabdoviridae*, *Hepeviridae*, deep transcriptome sequencing

## Abstract

*Macrosteles fascifrons*, a representative aster leafhopper frequently detected in rice-growing environments, is an economically significant insect that inhabits rice fields and plays a role in the ecology of crop pests and disease transmission. To expand the understanding of viral diversity associated with the aster leafhopper, we analyzed its virome using deep transcriptome sequencing. In addition to several previously reported viruses, we identified two previously unreported RNA viruses, tentatively designated as Macrosteles fascifrons hepe-like virus 1 (MfHV1) and Macrosteles fascifrons rhabdovirus 1 (MfRV1). The complete genome sequences of both genomes were obtained using overlapping RT-PCR and rapid amplification of cDNA ends. Excluding the poly(A) tail, the genome of MfHV1 is 6688 nucleotides in length and exhibits a genomic organization characteristic of the family *Hepeviridae*, comprising three major open reading frames (ORFs) that encode a putative nonstructural polyprotein, a capsid protein, and a small accessory protein. The ORF encoding the capsid protein partially overlaps with the ORF encoding the small accessory protein, a genomic feature commonly observed in hepe-like viruses. The genome of MfRV1 is 14,984 nucleotides in length and displays the canonical genomic organization of the family *Rhabdoviridae*. An additional accessory ORF was identified between the putative M and G genes. Phylogenetic analysis based on polyprotein sequences placed MfHV1 within the *Hepeviridae*, most closely related to insect-associated hepe-like viruses, whereas MfRV1 clustered within the subfamily *Deltarhabdovirinae*. According to ICTV guidelines, virus classification is based on a combination of sequence divergence, phylogenetic relationships, and genome organization. MfHV1 and MfRV1 share low amino acid sequence identities with known viruses (maximum 36.07% for the MfHV1 polyprotein and 47.7% for the MfRV1 RNA-dependent RNA polymerase). Based on sequence divergence, genome organization, and phylogenetic placement, these viruses are classified as putative novel members of their respective families. This study expands the diversity of virus-associated sequences detected in *M. fascifrons* and provides additional genomic resources for understanding insect-associated RNA viruses.

## Simple Summary

Leafhoppers are small insects commonly found in crop fields, and the diversity of viruses associated with many leafhopper species remains to be investigated. In this study, we examined the viruses present in the leafhopper *Macrosteles fascifrons*. Using high-throughput transcriptome sequencing, we analyzed viral genetic material within these insects and discovered two previously unknown RNA viruses. We obtained and analyzed the complete genetic sequences of both viruses and found that they are clearly different from any viruses reported before. These findings expand the current knowledge of the viral diversity associated with leafhoppers.

## 1. Introduction

Leafhoppers and planthoppers are widespread agricultural pests that cause significant crop losses through both direct feeding damage and the transmission of plant viruses [1]. Comprising more than 20,000 described species, these insects exploit a broad range of host plants, including cereals, legumes, fruit trees, vegetables, and ornamental crops. Viruses transmitted by these insects establish systemic infections in plants, resulting in symptoms such as stunting, chlorosis, and deformation [2]. Modern agricultural practices, particularly intensive monoculture with limited crop rotation or habitat diversification, can exacerbate virus spread through vector–virus interactions mediated by these insects. Therefore, a better understanding of leafhopper-associated viromes and the application of advanced molecular detection approaches may facilitate improved disease monitoring and management in agroecosystems [3].

Leafhoppers are recognized vectors of more than 150 plant viruses spanning diverse taxonomic groups, including members of the families *Reoviridae* (e.g., *Phytoreovirus*, *Fijivirus*), *Geminiviridae* (e.g., *Mastrevirus*), *Tombusviridae*, and *Caulimoviridae* (e.g., *Badnavirus*), as well as numerous unclassified viruses identified through high-throughput sequencing (HTS) [4,5,6,7,8]. Virus transmission by leafhoppers is typically persistent and propagative, whereby viruses circulate within the insect body and replicate in vector tissues such as the midgut, hemolymph, and salivary glands. This intimate association enables long-term transmission competence, allowing viruliferous insects to transmit viruses throughout their lifespan and thereby enhancing epidemic spread in agricultural systems [9]. The advent of HTS technologies has greatly accelerated virus discovery, enabling the unbiased detection of both known and novel viruses without prior sequence information [10,11]. These advances have revealed an unexpectedly high diversity of viruses associated with leafhoppers and raise important questions regarding virus evolution and potential cross-kingdom transmission between insects and plants.

The genus *Macrosteles* comprises small sap-feeding leafhoppers that are recognized as vectors of numerous plant pathogens, including viruses and phytoplasmas with significant agricultural impacts [12,13,14,15]. Among them, the aster leafhopper (*Macrosteles fascifrons*) is the primary vector of aster yellows phytoplasma, a pathogen responsible for severe diseases in a wide range of crops [13,14]. The movement of *Macrosteles* species among cultivated crops, adjacent fields, and surrounding vegetation—where they feed on grasses and weeds—facilitates pathogen dissemination and increases opportunities for acquisition and transmission by multiple vector populations [16]. Their high dispersal capacity and broad host range make *Macrosteles* species key drivers of pathogen spread across agroecosystems and critical targets for integrated pest and disease management strategies.

The family *Hepeviridae* comprises a group of positive-sense, single-stranded RNA viruses primarily infecting vertebrate hosts [17,18,19]. These viruses possess linear genomes of approximately 6.4–7.2 kb, featuring a 5′ cap structure and a 3′ poly(A) tail, and encoding three major open reading frames (ORFs) [19]. ORF1 encodes a multifunctional nonstructural polyprotein containing conserved domains, including methyltransferase, papain-like cysteine protease, helicase, and RNA-dependent RNA polymerase (RdRp). ORF2 encodes the capsid protein, and ORF3 encodes a small phosphoprotein involved in host interactions. Recent metagenomic studies have greatly expanded the diversity of hepevirus-related sequences, revealing numerous hepe-like viruses in invertebrates and environmental samples [20,21,22]. These viruses often form deeply divergent lineages basal to known members of *Hepeviridae*, suggesting an ancient evolutionary origin and a broader host range than previously appreciated.

The family *Rhabdoviridae* comprises a large and diverse group of enveloped viruses with non-segmented, negative-sense single-stranded RNA genomes [23]. Members of this family typically encode five canonical structural proteins—nucleoprotein (N), phosphoprotein (P), matrix protein (M), glycoprotein (G), and large polymerase protein (L)—in the conserved order 3′-N-P-M-G-L-5′, although additional accessory genes are frequently present [24]. Rhabdoviruses infect a broad range of hosts, including plants, vertebrates, and invertebrates, occupying diverse ecological niches in both terrestrial and aquatic environments [25,26]. Many animal-infecting rhabdoviruses are arthropod-borne, highlighting the central role of insects in their transmission cycles. Despite this diversity, rhabdoviruses generally exhibit host-associated phylogenetic clustering, suggesting relatively infrequent host-switching events. Their genomes are highly plastic, with frequent gene gain, loss, and rearrangement, contributing to substantial diversity in genome organization and complicating taxonomic classification [24].

In this study, we investigated the virome of *M. fascifrons* collected from rice fields using HTS. Comprehensive bioinformatic analyses revealed the presence of two previously reported viruses and two putatively novel viral species. We obtained the complete genome sequence of a putative novel hepe-like virus, designated Macrosteles fascifrons hepe-like virus 1 (MfHV1), with a genome of 6688 nt containing three canonical ORFs. Phylogenetic analysis based on the RdRp region placed MfHV1 within the family *Hepeviridae*, forming a distinct lineage most closely related to Sogatella furcifera hepe-like virus, supporting its classification as a putative novel member. In addition, we identified a putative novel rhabdovirus, designated Macrosteles fascifrons rhabdovirus 1 (MfRV1), with a genome length of 14,984 nt. Phylogenetic analysis based on the L protein sequences positioned MfRV1 within the subfamily *Deltarhabdovirinae*, and pairwise amino acid (aa) sequence identities of less than 50% relative to known viruses further support its designation as a new species. Collectively, these findings expand the current understanding of virus-associated diversity in leafhoppers and suggest that *M. fascifrons* may harbor diverse RNA viruses in rice agroecosystems.

## 2. Materials and Methods

### 2.1. Leafhopper Collection

Adult leafhoppers were captured from rice paddies in Jianning County (Fujian, China) in September 2025 using sweep nets. To ensure physiological stability, insects were briefly reared on virus-free rice seedlings. Individuals were subsequently maintained on healthy rice seedlings and identified under a stereomicroscope. Five *M. fascifrons* individuals were obtained and all were used for subsequent RNA extraction and sequencing. Samples were flash-frozen in liquid nitrogen and stored at −80 °C until further molecular analyses.

### 2.2. RNA Extraction and Library Preparation

Total RNA was extracted from a pool of five individuals using the TRIzol Reagent (Invitrogen, Carlsbad, CA, USA) following the manufacturer’s instructions. RNA concentration and purity were measured using a NanoDrop 2000 spectrophotometer (Thermo Scientific, Waltham, MA, USA), while RNA integrity was assessed by RNA-specific agarose gel electrophoresis and/or an Agilent 2100 Bioanalyzer (Agilent Technologies, Santa Clara, CA, USA). Ribosomal RNA (rRNA) was removed using the Epicentre Ribo-Zero™ rRNA Removal Kit (Illumina, San Diego, CA, USA) to reduce host rRNA background. The remaining RNA was fragmented using divalent cations at elevated temperature in Illumina fragmentation buffer and subsequently used for strand-specific cDNA library construction. Briefly, first-strand cDNA was synthesized using random oligonucleotide primers, followed by second-strand synthesis with DNA polymerase I using dUTP in place of dTTP. The resulting double-stranded cDNA was purified, end-repaired, A-tailed, and ligated to sequencing adapters. The second strand containing dUTP was selectively degraded using the USER enzyme (NEB, Ipswich, MA, USA). Library fragments of approximately 400–500 bp were size-selected using the AMPure XP system (Beckman Coulter, Brea, CA, USA), followed by PCR enrichment (15 cycles) with Illumina PCR Primer Cocktail. The library quality was assessed by the Agilent High Sensitivity DNA kit (Agilent Technologies, Santa Clara, CA, USA) on a Bioanalyzer 2100 system. The total library concentration was quantified using PicoGreen fluorescence assays (Quantifluor-ST fluorometer, Promega, Madison, WI, USA, E6090).

### 2.3. High Throughput Sequencing and Bioinformatic Analysis

HTS was performed on the XPLUS platform (Shanghai Personal Biotechnology Co., Ltd., Shanghai, China), generating 150 bp paired-end reads. Raw sequencing reads were quality-checked using FastQC (v0.12.1) and trimmed using fastp (v1.1.0) with parameters “-W 5 -M 20 -5 -3 -l 50” [27]. Clean reads were assembled de novo using Trinity (v2.15.1) with default parameters [28]. Assembled contigs were searched against a local viral protein database (NCBI Viral RefSeq, downloaded on 18 August 2025) using BLASTx (2.15.0) with an E-value cutoff of 1 × 10^−5^ to identify candidate viral sequences [29]. For each contig, only the best-scoring hit (top hit) was retained for downstream identification. Contigs with significant similarity to viral proteins with alignment lengths greater than 500 amino acids were selected as preliminary virus-associated candidates for virome diversity estimation. To improve the reliability of RNA virus identification and reduce potential false-positive assignments, candidate contigs were further curated based on viral taxonomic classification and protein annotation. Only sequences encoding RNA-dependent RNA polymerase (RdRP) or viral polyproteins were retained as high-confidence candidate RNA virus sequences for downstream analyses. Previously generated RNA-seq data from an *M. fascifrons* population collected in Yunxiao County (Fujian, China) were re-analyzed for comparative purposes in this study [5].

### 2.4. Confirmation and Determination of the Complete Viral Genome Sequence

To validate candidate viral sequences, specific primers were designed based on assembled contigs (Table S1). PCR amplification was performed using cDNA from total RNA. Amplicons were purified, cloned into the pMD19-T vector (Takara Bio, Kusatsu, Japan), and transformed into *Escherichia coli* (TransGen Biotech, Beijing, China) and verified by Sanger sequencing. In detail, the 5′ and 3′ terminal regions of MfHV1were obtained using the SMARTer^®^ RACE 5′/3′ Kit (Takara Bio, San Jose, CA, USA). For 3′ RACE, poly(A)+ RNA was reverse-transcribed using the 3′-RACE CDS Primer A, followed by PCR amplification using the Universal Primer Mix (UPM) and virus-specific primers. For 5′ RACE, first-strand cDNA synthesis was performed using the 5′-RACE CDS Primer A, and PCR amplification was carried out using the ISPCR primer in combination with virus-specific primers. For MfRV1, RNA was polyadenylated using *E. coli* Poly(A) Polymerase (New England Biolabs, Ipswich, MA, USA) before 3′ RACE.

### 2.5. Genome Annotation and Phylogenetic Analysis

ORFs were predicted using ORFfinder (https://www.ncbi.nlm.nih.gov/orffinder/, accessed on 11 November 2025) with a minimum length threshold of 300 nucleotides (nt). Protein sequences were annotated by BLASTp (2.15.0) against the NCBI non-redundant (nr) protein database with an E-value cutoff of 1 × 10^−5^. Conserved protein domains were identified using the NCBI Conserved Domain Database (CDD) (https://www.ncbi.nlm.nih.gov/Structure/cdd/wrpsb.cgi, accessed on 11 November 2025). RNA secondary structures were predicted using the RNAfold WebServer (http://rna.tbi.univie.ac.at//cgi-bin/RNAWebSuite/RNAfold.cgi, accessed on 11 November 2025) with default parameters based on minimum free energy models.

For comparative and phylogenetic analyses, proteins of MfHV1 and MfRV1 were aligned with representative sequences from the families *Hepeviridae* and *Rhabdoviridae*, respectively, using MAFFT (v7.310) with default parameters [30]. Maximum-likelihood (ML) phylogenetic trees were constructed using IQ-TREE (v3.0.1) [31], with substitution models selected by ModelFinder based on the Bayesian information criterion (BIC). Branch support was assessed with 1000 bootstrap replicates. Phylogenetic trees were visualized and annotated using MEGA X (v10.2) [32].

## 3. Results

### 3.1. Virome of M. fascifrons

To characterize the viral diversity associated with *M. fascifrons*, high-throughput transcriptome sequencing was performed. A total of 212 million paired-end raw reads were generated, corresponding to 31.64 Gb of high-quality sequence data. De novo assembly produced 572,811 contigs. Among these, 1557 contigs showed significant similarity to proteins of 107 viral taxa (alignment length > 500 aa). These were retained as candidate virus-associated sequences (Table S2). Those sequences were further assigned to a broad range of viral taxa spanning multiple families, including *Phycodnaviridae* (*n* = 19), *Mimiviridae* (*n* = 13), *Poxviridae* (*n* = 11), *Baculoviridae* (*n* = 8), and *Iridoviridae* (*n* = 4), among others (Figure S1A). For comparison, analysis of previously generated RNA-seq data from *M. fascifrons* collected in Yunxiao, Fujian Province, China (June 2021), identified 960 contigs with similarity to proteins from 89 putative viral taxa (Table S3) [5]. Across the two datasets, 76 putative viral taxa were detected in samples from both geographic regions (Figure S1B,C), indicating partial overlap as well as differences in virus-associated sequence composition.

From these candidates, 24 isoforms (derived from eight contigs) showed the best matches to viral polyproteins or RdRps, suggesting the presence of diverse associated RNA virus–related sequences detected in the insect samples. A 10,308-nt contig shared 46.37% aa identity with the polyprotein of Psammotettix alienus iflavirus 1 (NC_040724.1, genome length: 10,836 nt). This virus, designated Macrosteles fascifrons iflavirus 2 (MfIF2), contains a single ORF encoding a 3118-aa polyprotein and shares 97.11% amino acid identity with a previously reported iflavirus from *M. fascifrons*, supporting it as an isolate of MfIV1 [5]. A 7639-nt contig exhibited the highest similarity to the RdRp of Spissistilus festinus virus 1 (NC_014359.1, genome length: 7951 nt), sharing 56% amino acid identity with 93% query coverage. Given its sequence identity relative to commonly observed species demarcation levels, the taxonomic status of this virus remains uncertain, and it may represent either a new species or a divergent isolate of a previously described virus.

Notably, a 15,019-nt contig exhibited significant similarity to the RdRp of Diachasmimorpha longicaudata rhabdovirus (NC_030451.1, genome length: 13,434 nt), sharing 58% aa identity. According to the species demarcation criteria established by the International Committee on Taxonomy of Viruses (ICTV, https://ictv.global/report/rhabdoviridae, accessed on 29 April 2026), new rhabdoviruses are generally distinguished based on less than 80% sequence identity of the L protein, together with phylogenetic relationships and genome organization. In this context, the observed sequence divergence suggests the presence of a previously uncharacterized rhabdovirus associated with *M. fascifrons*. This virus was therefore designated Macrosteles fascifrons rhabdovirus 1 (MfRV1). Similarly, a 6699-nt contig shared 36.06% aa identity with the polyprotein of Sogatella furcifera hepe-like virus (NC_040710.1, genome length: 7312 nt). The relatively low sequence identity, together with its first detection in *M. fascifrons*, suggests the presence of a putative novel hepe-like virus, provisionally named Macrosteles fascifrons hepe-like virus 1 (MfHV1).

### 3.2. Genome Organization and Annotation of MfHV1

**Figure 1:**
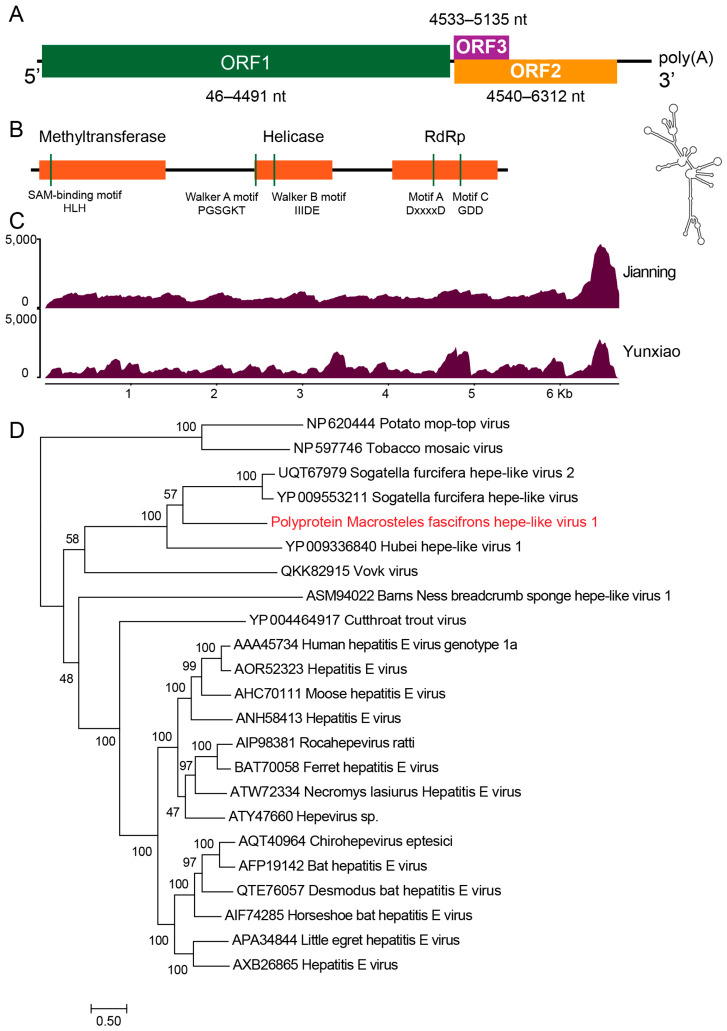
Genome organization and molecular characterization of MfHV1. (**A**) Schematic representation of the MfHV1 genome (6688 nt, excluding poly(A) tail), comprising three open reading frames (ORFs). The 375-nt 3′UTR has a high GC content (~58%) and forms a stable RNA secondary structure. (**B**) Conserved domain architecture of the ORF1-encoded polyprotein, including methyltransferase (Mtr), helicase (Hel), and RNA-dependent RNA polymerase (RdRp) domains. Characteristic conserved motifs are indicated, including the SAM-binding motif (H-L-H) in the methyltransferase, Walker A (PGSGKT) and Walker B (IIDDE) motifs in the helicase, and motifs A (DxDxxD) and C (GDD) in the RdRp. (**C**) Distribution of sequencing read coverage across the MfHV1 genome in *M. fascifrons* samples from Jianning and Yunxiao. Coverage profiles were generated by mapping clean reads to the assembled genome, showing overall support for genome continuity with variation in read depth along the genome. (**D**) Maximum-likelihood phylogenetic tree based on complete polyprotein sequences of MfHV1 and 20 representative hepe-like viruses, with Potato mop-top virus and Tobacco mosaic virus used as outgroups. MfHV1 identified in this study is highlighted in red. The tree was inferred using IQ-TREE under the best-fit substitution model (LG + F + I + G), selected according to the Bayesian information criterion (BIC), with 1000 bootstrap replicates. Bootstrap support values (%) are indicated at the nodes, and the scale bar represents amino acid substitutions per site.

Excluding the poly(A) tail, the complete genome of MfHV1 is 6688 nt in length with an overall GC content of 57% (GenBase accession: C_AA160127.1, Figure S2A). The genome contains three predicted ORFs and is flanked by a 53-nt 5′ untranslated region (UTR) and a 375-nt 3′UTR. The 375-nt 3′UTR exhibits a relatively high GC content (~58%) with stable RNA secondary structure. Its genomic organization follows the canonical arrangement 5′UTR–ORF1–ORF2/ORF3–3′UTR, which is characteristic of hepe-like viruses (Figure 1).

ORF1 (nt 45–4491) represents the largest coding region and encodes a 1481-aa polyprotein. Online BLASTp analysis revealed that this protein shares the highest similarity with the replication polyprotein of Sogatella furcifera hepe-like virus (YP_009553211.1). Conserved domain analysis identified the typical replication-associated domains, including a viral methyltransferase domain (PF01660; aa 26–417), a superfamily RNA helicase domain (PF01443; aa 694–934), and an RdRp domain (PF00978; aa 1122–1448) (Figure 1B). Within the helicase region, the conserved Walker A motif (PGSGKT) and Walker B motif (IIIDE) were detected, consistent with ATP-dependent helicase activity. The RdRp contains conserved catalytic motifs typical of positive-sense RNA viruses, including motif A (DxxxxD) and motif C (GDD) [33]. In addition, a conserved SGE motif was detected within the RdRp region, which may contribute to maintaining catalytic function. Downstream of ORF1, ORF2 (nt 4543–6315) encodes a 590-aa protein and partially overlaps with ORF3 (nt 4536–5138), which is located in an alternative reading frame. ORF2 is predicted to encode the major capsid protein (CP) and shows the highest sequence similarity to the CP of Sogatella furcifera hepe-like virus 2 (UQT67980.1). Domain analysis identified a DiSB-ORF2_chro domain (PF16506; aa 304–351), associated with putative virion glycoproteins in insect viruses [34]. Coverage is generally uniform across the genome in the Jianning sample. Notably, it was also detected in *M. fascifrons* from Yunxiao, indicating its presence across multiple geographic locations (Figure 1C).

### 3.3. MfHV1 Represents a Putative Novel Member of the Family Hepeviridae

To determine the evolutionary relationship of MfHV1 in the family *Hepeviridae*, phylogenetic analysis was performed based on the amino acid sequence of the ORF1 polyprotein, including representative viruses from this family (Figure 1D). The resulting maximum-likelihood tree showed that MfHV1 forms a well-supported clade with insect-associated hepe-like viruses, clustering most closely with Sogatella furcifera hepe-like virus [17,35]. This placement indicates that MfHV1 belongs to the insect-associated hepe-like virus lineage within the family *Hepeviridae*.

**Figure 2:**
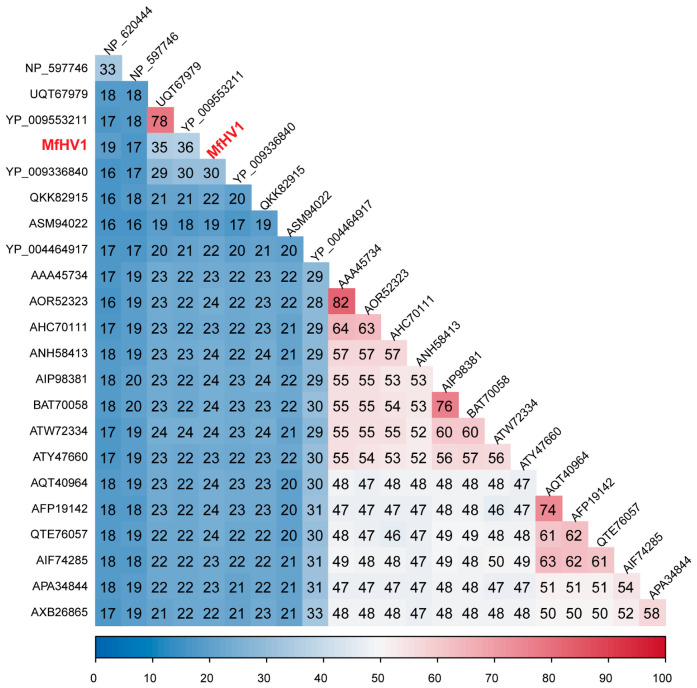
Pairwise amino acid identity analysis of MfHV1 and representative hepe-like viruses. Heatmap showing pairwise amino acid sequence identities (%) calculated from the aligned RdRp region of MfHV1 and representative members of the family *Hepeviridae*. Multiple sequence alignments were generated using MAFFT, and identity values were computed from the full-length aligned RdRp regions. MfHV1 identified in this study is highlighted in red. Accession numbers of the sequences used in the phylogenetic analysis are indicated next to the corresponding taxa. Color scale represents pairwise amino acid sequence identity (%), with values ranging from 0 (blue) to 100 (red).

Furthermore, pairwise sequence comparisons among insect-associated hepe-like viruses further supported this relationship (Figure 2). MfHV1 shares only 36.07% identity in the polyprotein with Sogatella furcifera hepe-like virus, while identities with other representative hepe-like viruses are generally lower (approximately 16–33%). This level of sequence divergence falls well below the threshold typically used to demarcate species within the family *Hepeviridae* [19]. Taken together, the low sequence identity, its distinct phylogenetic position, and its occurrence in a previously unreported insect sample are consistent with MfHV1 representing a putative novel member of the insect-associated hepe-like viruses.

### 3.4. Characterization of a Putative Novel Virus in the Family Rhabdoviridae

**Figure 3:**
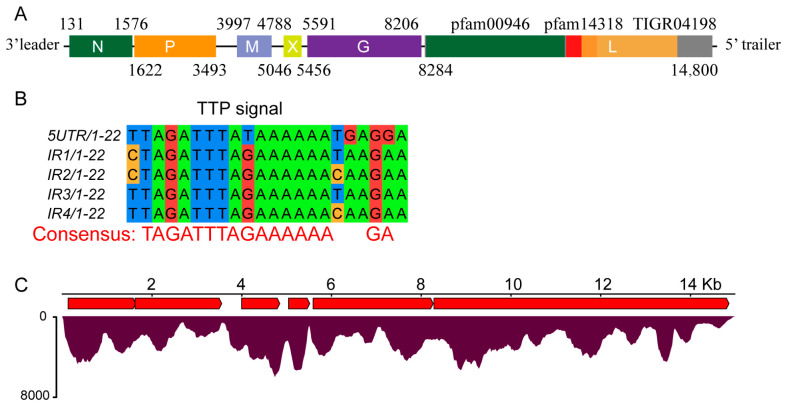
Genome organization and transcriptional features of MfRV1. (**A**) Schematic representation of the MfRV1 genome, organized in the canonical order 3′-leader–N–P–M–X–G–L–trailer-5′. ORFs are shown as colored boxes with genomic coordinates indicated above and below each gene. Conserved protein domains identified in the L protein, including motifs corresponding to RNA-dependent RNA polymerase and associated functional regions (e.g., pfam00946, pfam14318, TIGR04198), are indicated. (**B**) Sequence features of gene junction regions, showing conserved transcription termination/polyadenylation (TTP) signals and short intergenic regions. The alignment highlights a conserved TTP motif (poly-U tract) followed by a conserved intergenic dinucleotide (GA). The consensus sequence derived from multiple junctions is shown below, indicating conserved elements involved in transcription termination and reinitiation. (**C**) RNA-seq read coverage across the MfRV1 genome based on mapped sequencing reads. The coverage profile illustrates read distribution along the genome, supporting the assembly and indicating variation in transcript abundance across different genomic regions.

The complete genome of MfRV1 (GenBase accession: C_AA160128.1, Figure S2B) is 14,984 nt in length and consists of a single-stranded negative-sense RNA genome organized in the canonical 3′–5′ orientation characteristic of insect-associated members of the family *Rhabdoviridae* (Figure 3). The genome contains a 104-nt 5′ UTR and a 130-nt 3′ UTR. MfRV1 contains six major ORFs separated by short intergenic regions, arranged in the order 3′-leader–N–P–P1–M–G–L–trailer-5′ on the antigenomic strand. In MfRV1, ORF1 (1446 nt), ORF2 (1872 nt), ORF3 (792 nt), ORF5 (2616 nt), and ORF6 (6597 nt) encode the five conserved structural proteins of rhabdoviruses—N, P, M, G, and L, respectively. In addition to these canonical genes, an accessory ORF (ORF4, 411 nt) is located between the putative M and G genes and encodes a small protein with no significant similarity to sequences in public databases. Conserved domain analysis of the L protein identified a Mononeg_RNA_pol domain (pfam00946, aa 12–1070, E-value = 7.68 × 10^−120^), corresponding to the catalytic core responsible for RNA synthesis. Downstream regions include a Mononeg_mRNAcap domain (pfam14318, aa 1082–1318, E-value = 2.91 × 10^−44^), and a paramyx_RNAcap domain (TIGR04198, aa 1182–2035, E-value = 1.86 × 10^−38^). The L protein also contains conserved motifs characteristic of mononegaviral polymerases, including the GDNQ motif associated with RdRP activity and the HR motif involved in PRNTase-mediated mRNA capping, as well as residues typical of the S-adenosyl-L-methionine (SAM)-dependent methyltransferase region.

Analysis of intergenic regions revealed conserved gene transcription termination/polyadenylation (TTP) signals at the end of each viral gene (Figure 3B), consistent with the canonical stop–start transcription mechanism of rhabdoviruses. Notably, the first TTP signal is located within the coding region of ORF2 rather than at the intergenic junction. A conserved intergenic dinucleotide (GA) was also identified at gene junctions. Analysis of transcription patterns based on sequencing data showed no clear transcriptional gradient. This profile suggests the occurrence of transcriptional read-through during viral RNA synthesis, potentially contributing to the production of full-length complementary antigenomic RNA (Figure 3C).

### 3.5. MfRV1 Is a Putative New Member Belonging to the Family Rhabdoviridae

**Figure 4:**
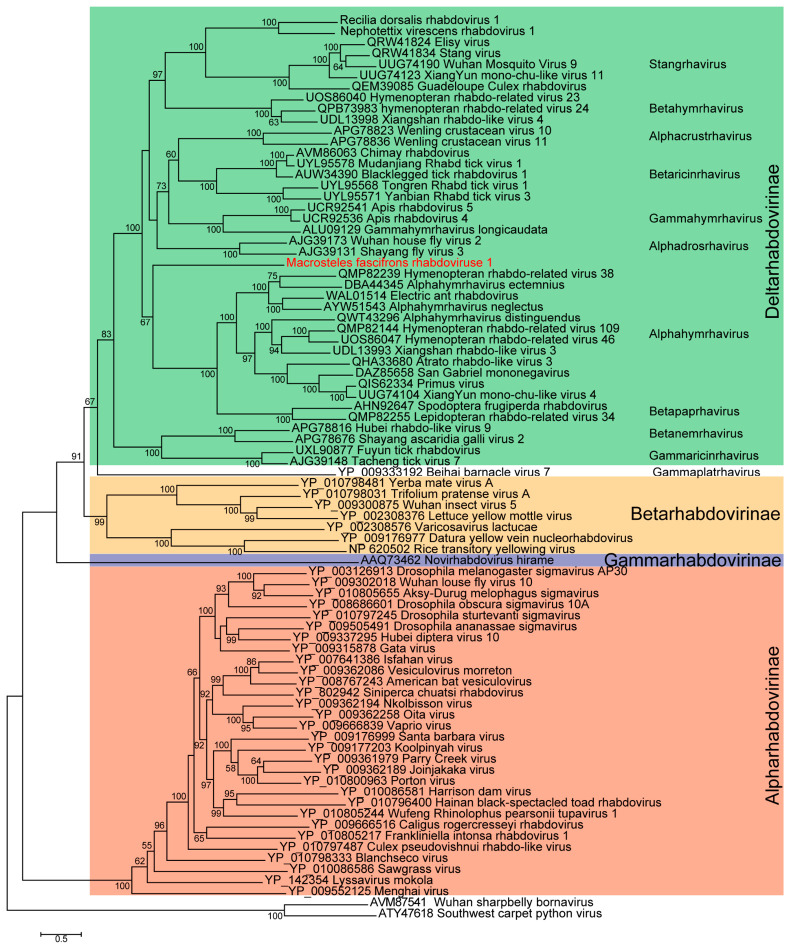
Phylogenetic analysis of MfRV1 within the family *Rhabdoviridae*. Maximum-likelihood phylogenetic tree inferred from complete amino acid sequences of the L proteins of MfRV1 and 79 representative members of the family *Rhabdoviridae*, including representatives of the subfamilies *Alpharhabdovirinae* (salmon), *Betarhabdovirinae* (orange), *Gammarhabdovirinae* (purple), and 38 members of the subfamily *Deltarhabdovirinae* (green), as well as two previously reported rhabdovirus-like sequences identified in our previous study. Multiple sequence alignment was performed using MAFFT, and the ML tree was constructed using IQ-TREE under the best-fit substitution model (Q.PFAM + F + R8), selected according to the BIC. Branch support was assessed with 1000 bootstrap replicates, and only values ≥ 50% are shown at the nodes. The scale bar represents the number of amino acid substitutions per site.

To determine the evolutionary placement of MfRV1 within the family *Rhabdoviridae*, a maximum-likelihood phylogenetic analysis was performed based on the L protein, including representative members of the subfamilies *Alpharhabdovirinae*, *Betarhabdovirinae*, and *Gammarhabdovirinae*, and 38 members of the subfamily *Deltarhabdovirinae*, along with two previously reported novel viruses [6]. The resulting phylogeny resolved the four major subfamilies, with MfRV1 robustly clustering within *Deltarhabdovirinae*, supporting its assignment to this subfamily (Figure 4). Within this clade, MfRV1 groups with insect-associated rhabdoviruses but forms a distinct and well-supported branch, separate from previously described genera.

Pairwise aa sequence comparisons of the L protein further supported this placement (Figure S3). Overall, MfRV1 exhibits relatively low sequence identity to most representative rhabdoviruses, consistent with its position on a distinct branch in the phylogenetic tree. The highest identity observed was 47.7% with the RdRp of Diachasmimorpha longicaudata rhabdovirus (DlonRVa; ALU09129.1). Although MfRV1 clusters with insect-associated members within *Deltarhabdovirinae*, its sequence identity remains modest compared to that observed among closely related viruses within established groups, indicating substantial sequence divergence. In contrast, higher identity values are observed among viruses within the same clusters, further highlighting the separation of MfRV1 from these lineages. Consistently, BLASTp analysis against the NCBI nr database identified the closest homolog of the MfRV1 L protein as the polyprotein of Hemipteran rhabdo-related virus OKIAV30 (QMP81799.1), with 45.15% amino acid identity. Taken together, the phylogenetic placement of MfRV1 within *Deltarhabdovirinae*, its separation from established lineages, and its moderate sequence identity to known viruses support its classification as a distinct lineage among insect-associated rhabdoviruses.

## 4. Discussion

Leafhoppers are widely recognized as important vectors of plant and animal viruses, playing critical roles in the epidemiology of numerous agricultural diseases [12]. Characterizing the virome of these insect vectors is therefore essential for predicting potential viral threats to host plants across diverse ecological contexts [11,16]. In this study, we investigated the virome of *Macrosteles fascifrons* collected from rice fields and identified a diverse assemblage of RNA viruses. In addition to a previously reported MfIF1 and a totivirus-like sequence [5], we identified two new viruses, a hepe-like virus (MfHV1) and a rhabdovirus (MfRV1). Complete genome characterization and phylogenetic analyses indicate that both viruses represent distinct viral lineages associated with *M. fascifrons*.

Comparative analysis of virus-associated sequences detected in *M. fascifrons* from Jianning and Yunxiao revealed both shared and distinct viral components (Figure S1) [5]. At the family level, similar major viral groups were identified in both populations, while differences were observed in the composition and representation of virus-like taxa. A total of 76 viral taxa were detected in both datasets, whereas 31 and 13 taxa were unique to the Jianning and Yunxiao samples, respectively (Figure S1C), indicating partial overlap in virus-associated sequence composition between the two locations. Among the identified viruses, an iflavirus was detected in both populations, consistent with its presence across datasets. MfHV1 was also identified in both Jianning and Yunxiao samples, suggesting that this hepe-like virus is not restricted to a single location. In contrast, MfRV1 was detected only in the Jianning dataset. This difference may reflect spatial or temporal variation, differences in sampling and sequencing conditions, or stochastic detection of low-abundance viruses, rather than a definitive geographic restriction [36]. Beyond presence–absence variation, differences in read representation were observed. For example, MfTV1 accounted for 18,853 paired-end reads in the Jianning library, compared to a substantially lower 584 reads in the Yunxiao samples. These variations may be influenced by multiple factors, including local environmental conditions, host population structure, and complex interactions within local microbial and plant communities [37]. However, given the limited sampling and differences in experimental design, these observations should be interpreted cautiously. Our findings highlight that insect virome data are highly sensitive to spatial and temporal context. Future studies employing longitudinal and multi-regional sampling are required to establish the infection stability of these viruses versus transient, environmentally mediated occurrences.

The identification of MfHV1 substantially expands the known host range of the family *Hepeviridae*, a group previously characterized as primarily vertebrate-infecting [19]. While historical associations centered on vertebrates, recent meta-transcriptomic surveys have uncovered an expansive diversity of hepe-like viruses across invertebrate taxa [17,18,21,35,38,39,40]. The genomic features of MfHV1—specifically its three-ORF structure and conserved MTase, Hel, and RdRp domains—align with other insect-associated hepe-like viruses, indicating a stable evolutionary core [41]. The phylogenetic placement of MfHV1 within an insect-specific clade reinforces the existence of a distinct evolutionary lineage within the *Hepelivirales*. Given their immense ecological diversity and multi-trophic interactions, insects likely represent a significant reservoir for viral diversification. Although these insect-associated lineages may offer critical insights into the evolutionary trajectory of the *Hepeviridae*, extensive sampling is required to elucidate the ancestral relationships and potential cross-taxa connections with vertebrate-infecting counterparts.

Genomic characterization of MfRV1 revealed features typical of arthropod-associated rhabdoviruses, including an accessory ORF situated between the P and M genes [23]. While such accessory genes are widely documented in the *Rhabdoviridae*, their functional roles in virus–host dynamics remain largely elusive [42]. Notably, a conserved TTP signal was identified internally within the ORF2 coding sequence rather than at a standard intergenic junction. This atypical architecture suggests non-canonical transcriptional regulation, potentially facilitating alternative outcomes such as premature termination or read-through. Supporting this, the absence of a discernible transcriptional gradient across the MfRV1 genome indicates a departure from the classical transcriptional attenuation model. While these features may provide enhanced transcriptional plasticity, their biological implications warrant further experimental validation.

Overall, our findings expand the known viral landscape of virus-associated sequences detected in *M. fascifrons* and highlight the complexity of insect-associated viromes. Differences observed between populations suggest that local ecological and environmental factors may influence the composition of virus-associated communities. It should be noted that these findings are based on sequence similarity and do not provide direct evidence of viral replication or stable host association. Given the status of leafhoppers as critical agricultural vectors, further characterization of their viromes may provide useful insights into viral diversity and evolution. Future research integrating longitudinal geographic surveys and functional assays will be pivotal in elucidating the transmission dynamics and fitness impacts of these viruses within complex agroecosystems.

## Data Availability

The original contributions presented in the study are included in the article/Supplementary Materials. The raw sequence data from this study are available in the Genome Sequence Archive (GSA: CRA039679) at the National Genomics Data Center (NGDC, https://ngdc.cncb.ac.cn/gsa, accessed on 1 May 2026). Further inquiries can be directed to the corresponding authors.

## Supplementary Materials

The following supporting information can be downloaded at: https://www.mdpi.com/article/10.3390/insects17050479/s1. Figure S1: Diversity of virus-associated sequences detected in *M. fascifrons* from Jianning and Yunxiao. (A–B) Distribution of virus-like taxa at the family level identified in the Jianning (A) and Yunxiao (B) datasets. The number of taxa assigned to each viral family is shown on the x-axis. (C) Venn diagram showing the overlap of virus-associated taxa between Jianning and Yunxiao populations. The number and percentage of shared and unique taxa are indicated for each dataset. Figure S2: Experimental validation of viral genome assembly by RT-PCR and RACE. (A) Validation of the MfHV1 genome. Schematic diagram of contig assembly and primer design for RT-PCR and RACE (top). The complete genome was confirmed by amplifying overlapping fragments (S1–S9) and terminal regions using 5′ and 3′ RACE. Genome continuity was confirmed by amplification of overlapping fragments (S1–S9) using primer pairs listed in Table S1 (MfHV1_S1–S9), together with terminal regions obtained by 5′ and 3′ RACE. Agarose gel electrophoresis (bottom) shows the corresponding amplification products. Lane “Marker” indicates DNA size standards. Fragment positions are indicated below the gel (S1: 160–811 nt; S2: 757–1806 nt; S3: 1258–2169 nt; S4: 1710–3218 nt; S5: 3179–4094 nt; S6: 3452–4450 nt; S7: 4128–5058 nt; S8: 4525–5768 nt; S9: 5692–6397 nt); (B) Validation of the MfRV1 genome. Schematic representation of contig assembly and primer positions used for RT-PCR and RACE amplification (top). Contigs were joined based on overlapping regions, and terminal sequences were obtained using 5′ and 3′ rapid amplification of cDNA ends (RACE). Genome assembly was confirmed by amplification of overlapping fragments (S1–S12) using primer pairs listed in Table S1 (MfRV1_S1–S12) Agarose gel electrophoresis (bottom) shows amplification products for overlapping fragments (S1–S12) spanning the complete genome. Lane “Marker” indicates DNA size standards. The positions and expected sizes of each fragment are indicated below the gel (S1: 118–1118 nt; S2: 1094–2510 nt; S3: 2480–3920 nt; S4: 3886–5176 nt; S5: 5156–6383 nt; S6: 6199–7621 nt; S7: 7600–8954 nt; S8: 8930–10315 nt; S9: 10286–11491 nt; S10: 11383–12963 nt; S11: 12819–13685 nt; S12: 13664–14789 nt); Figure S3: Pairwise amino acid identity of MfRV1 and representative rhabdoviruses based on the L protein, which was calculated based on pairwise amino acid alignments using MAFFT. Table S1: Primers used in this study. Primers used for amplification and validation of MfHV1 and MfRV1 genomes. Primer names, nucleotide sequences (5′–3′), and corresponding annotations are provided. Primers labeled as “S1–S12” represent overlapping regions used for RT-PCR amplification to confirm genome continuity. Primers with 5′ and 3′ prefixes were used for rapid amplification of cDNA ends (RACE) to determine terminal sequences. Forward (F) and reverse (R) primers are indicated accordingly. Table S2: Summary of candidate virus-associated contigs identified from the *M. fascifrons* transcriptome from Jianning. This table lists contigs showing significant similarity to viral proteins based on BLASTx searches against the NCBI Viral RefSeq database. For each contig, the best-scoring hit is reported, including accession number, percentage amino acid identity, alignment length, number of mismatches and gaps, query and subject alignment coordinates, E-value, and bit score. The predicted protein type (e.g., polyprotein or RNA-dependent RNA polymerase) and the corresponding virus species annotation are also provided. Only contigs with alignment lengths greater than 500 amino acids were retained as candidate virus-associated sequences for further analysis. Table S3: Summary of candidate virus-associated contigs identified from re-analysis of the Yunxiao *M. fascifrons* transcriptome dataset.
