# Supplementary material for: Genome Characterization of a Novel Hepe-like Virus and a Rhabdovirus Identified in Macrosteles fascifrons
Source: Insects. 2026 May 8;17(5):479. doi: 10.3390/insects17050479 (PMC13207742; doi:10.3390/insects17050479)
Supplement: Supplementary file 1 [file insects-17-00479-s001.zip › Figure S1.pdf]

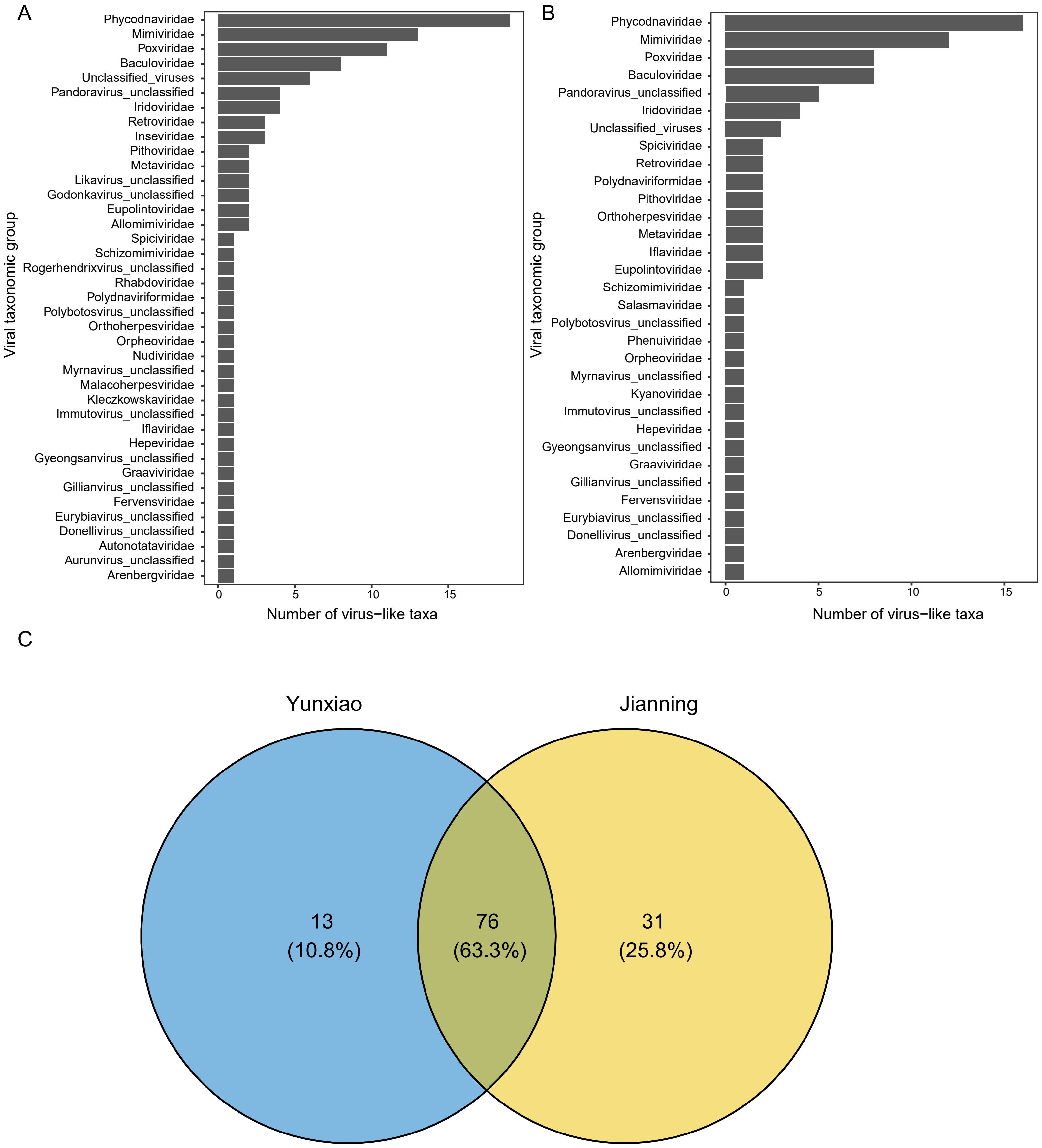

Figure S1: Diversity of virus-associated sequences detected in M. fascifrons from Jianning and Yunxiao. (A–B) Distribution of virus-like taxa at the family level identified in the Jianning (A) and Yunxiao (B) datasets. The number of taxa assigned to each viral family is shown on the x-axis. (C) Venn diagram showing the overlap of virus-associated taxa between Jianning and Yunxiao pop-ulations. The number and percentage of shared and unique taxa are indicated for each dataset.
