# Supplementary material for: Genome Characterization of a Novel Hepe-like Virus and a Rhabdovirus Identified in Macrosteles fascifrons
Source: Insects. 2026 May 8;17(5):479. doi: 10.3390/insects17050479 (PMC13207742; doi:10.3390/insects17050479)
Supplement: Supplementary file 1 [file insects-17-00479-s001.zip › Figure S2.pdf]

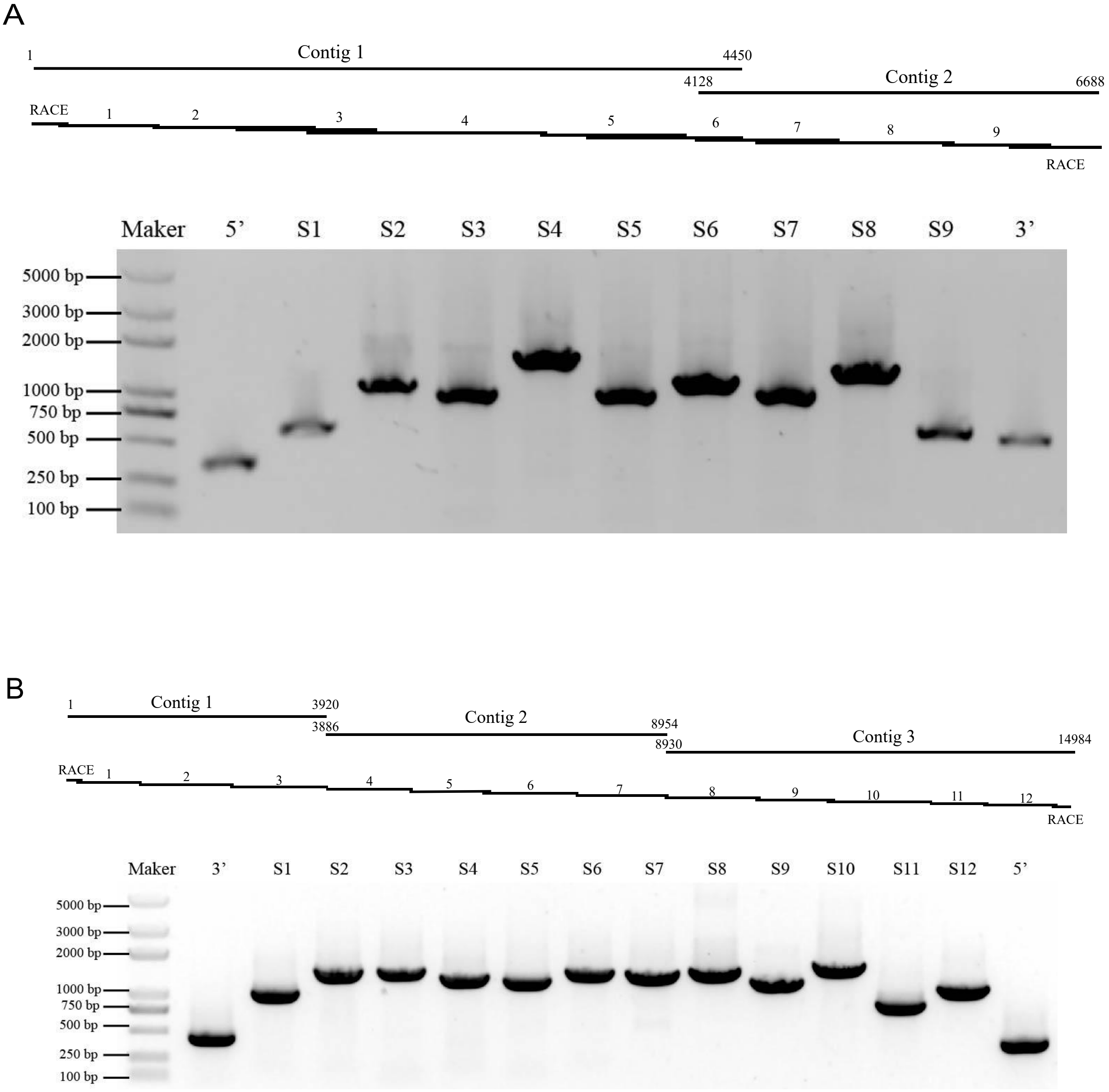

Figure S2: Experimental validation of viral genome assembly by RT-PCR and RACE. (A) Vali-dation of the MfHV1 genome. Schematic diagram of contig assembly and primer design for RT-PCR and RACE (top). The complete genome was confirmed by amplifying overlapping fragments (S1–S9) and terminal regions using 5′ and 3′ RACE. Genome continuity was confirmed by amplification of overlapping fragments (S1–S9) using primer pairs listed in Table S1 (MfHV1_S1–S9), together with terminal regions obtained by 5′ and 3′ RACE. Agarose gel electrophoresis (bottom) shows the corresponding amplification products. Lane “Marker” indicates DNA size standards. Fragment positions are indicated below the gel (S1: 160–811 nt; S2: 757–1806 nt; S3: 1258–2169 nt; S4: 1710–3218 nt; S5: 3179–4094 nt; S6: 3452–4450 nt; S7: 4128–5058 nt; S8: 4525–5768 nt; S9: 5692–6397 nt); (B) Validation of the MfRV1 genome. Schematic representation of contig assembly and primer po-sitions used for RT-PCR and RACE amplification (top). Contigs were joined based on overlapping regions, and terminal sequences were obtained using 5′ and 3′ rapid amplification of cDNA ends (RACE). Genome assembly was confirmed by amplification of overlapping fragments (S1–S12) using primer pairs listed in Table S1 (MfRV1_S1–S12) Agarose gel electrophoresis (bottom) shows amplification products for overlapping fragments (S1–S12) spanning the complete genome. Lane “Marker” indicates DNA size standards. The positions and expected sizes of each fragment are indicated below the gel (S1: 118–1118 nt; S2: 1094–2510 nt; S3: 2480–3920 nt; S4: 3886–5176 nt; S5: 5156–6383 nt; S6: 6199–7621 nt; S7: 7600–8954 nt; S8: 8930–10315 nt; S9: 10286–11491 nt; S10: 11383–12963 nt; S11: 12819–13685 nt; S12: 13664–14789 nt);
