# Supplementary figures and images for: Genome Characterization of a Novel Hepe-like Virus and a Rhabdovirus Identified in Macrosteles fascifrons
Source: Insects. 2026 May 8;17(5):479. doi: 10.3390/insects17050479 (PMC13207742; doi:10.3390/insects17050479)

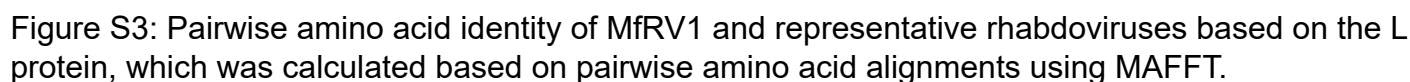

Supplement: Supplementary file 1 [file insects-17-00479-s001.zip › Figure S3.pdf]
